# Massive endocytosis mechanisms are involved in uptake of HIV-1 particles by monocyte-derived dendritic cells

**DOI:** 10.3389/fimmu.2024.1505840

**Published:** 2025-01-10

**Authors:** Fernando Laguía, Jakub Chojnacki, Itziar Erkizia, María Isabel Geli, Carlos Enrich, Javier Martinez-Picado, Patricia Resa-Infante

**Affiliations:** ^1^ IrsiCaixa, Badalona, Spain; ^2^ CIBERINFEC, Madrid, Spain; ^3^ Germans Trias i Pujol Research Institute (IGTP), Badalona, Spain; ^4^ Department of Cell Biology, Institute for Molecular Biology of Barcelona (IBMB, CSIC), Barcelona, Spain; ^5^ Cell Compartments and Signaling Group, Institut d’Investigacions Biomediques August Pi i Sunyer (IDIBAPS), Barcelona, Spain; ^6^ Departament de Biomedicina, Facultat de Medicina i Ciències de la Salut, Universitat de Barcelona, Barcelona, Spain; ^7^ University of Vic-Central University of Catalonia (UVic-UCC), Vic, Spain; ^8^ Catalan Institution for Research and Advanced Studies (ICREA), Barcelona, Spain

**Keywords:** dendritic cells, CD169/Siglec1, HIV, sac-like compartment, MEND

## Abstract

**Introduction:**

HIV-1 exploits dendritic cells (DCs) to spread throughout the body via specific recognition of gangliosides present on the viral envelope by the CD169/Siglec-1 membrane receptor. This interaction triggers the internalization of HIV-1 within a structure known as the sac-like compartment. While the mechanism underlying sac-like compartment formation remains elusive, prior research indicates that the process is clathrin-independent and cell membrane cholesterol–dependent and involves transient disruption of cortical actin. Here, we investigate the potential involvement of massive endocytosis (MEND) in this process.

**Methods:**

We used live cell confocal imaging to measure the dimensions and dynamics of the compartment. We assessed the role of actin and cholesterol in fixed and live cells using confocal microscopy and evaluated the effect of PI3K and protein palmytoilation inhibitors during viral uptake.

**Results:**

Our data demonstrate extensive plasma membrane invagination based on sac-like compartment dimensions (2.9 μm in diameter and 20 μm^3^ in volume). We showed that the cholesterol concentration doubles within the regions of viral uptake, suggesting lipid-phase separation, and that development of the sac-like compartment is accompanied by transient depolarization of cortical actin. Moreover, we observed that protein palmitoylation and PI3K inhibition reduce the sac-like compartment formation rate from 70% to 20% and 40%, respectively.

**Conclusions:**

Our results indicate the involvement of MEND mechanisms during sac-like compartment formation.

## Introduction

Antigen-presenting cells of the myeloid lineage, including monocytes, macrophages, and dendritic cells (DCs), initiate immune responses and are crucial for inducing resistance to invading viruses. DCs patrol peripheral tissues and are among the first cells to interact with incoming viruses, enabling capture, processing, and antigen presentation. However, some viruses, such as the human immunodeficiency virus (HIV-1), take advantage of myeloid cell activity to facilitate viral dissemination in a *trans*-infection process. Thus, captured viral particles are transferred to target CD4^+^ T cells located in secondary lymphoid tissue, with no productive infection in the myeloid cell (1–3).

The sialic acid–binding Ig-like lectin 1 (Siglec-1/CD169) transmembrane protein is a cell adhesion molecule that mediates HIV-1 uptake in DCs and macrophages. This myeloid receptor is expressed in an inflammation-dependent manner following activation with type-I interferon and recognizes sialic acid molecules in gangliosides such as GM-1 and GM-3, which are incorporated into the envelope of HIV particles as they bud from the plasma membrane of infected cells (4–10). Upon binding of HIV-1 particles to CD169, the receptor polarizes towards a cellular pole and traps the viral particles within a sac-like compartment (5, 11, 12). In this structure, viral particles are partially protected from degradation and remain connected to the extracellular space facilitating *trans*-infection (11, 13, 14). This phenomenon has been demonstrated in several cellular models, including mature monocyte-derived dendritic cells (MDDCs), mature monocyte-derived macrophages (MDMs), and CD169^+^ CD11c^+^ BDCA1^+^ *ex-vivo* cells from tonsils (15). In myeloid cells obtained *ex-vivo* from dermal tissues, CD169-dependent binding to HIV-1 and *trans*-infection of CD4^+^ T cells have been observed, particularly in MDMs, but also partially in MDDCs (16, 17). Moreover, sac-like compartments have been detected *in vivo* in DCs from infected vaginal tissue (2). These compartments display similarities to the virus-containing compartments (VCCs) described in macrophages, which are also formed upon interaction between CD169 and HIV-1 and accumulate *de novo* synthesized viral particles (18–20).

While viruses can utilize different endocytosis mechanisms for replicating in the host cell (21), CD169-dependent viral entry does not lead to productive infection (6). Information on the initiation of endocytic cascade by this receptor has been limited, particularly considering the absence of a tyrosine-based activation motif in the CD169 cytosolic domain (22). Viral particles bind to the cell membrane of MDDCs and coalesce on the cell surface before being internalized and accumulating in the sac-like compartment. Similarly, the mechanism behind viral particle migration and sac-like compartment formation remains unclear. While cholesterol sequestration and cytoskeleton disruption both prevent accumulation of HIV-1 in the sac-like compartment (1, 23), the mechanism triggering these processes is not well understood. Filamentous actin regulators, such as ERM complex and formin, need to be inactivated immediately after viral binding to enable CD169 nanoclustering and virus polarization in the cell membrane at very early stages of sac-like compartment formation, indicating the requirement of major cortical actin rearrangement during sac-like compartment formation (23). In turn, inhibition of clathrin-mediated endocytosis does not affect the process (14, 24). The conclusions reached above suggest that an unknown specific signaling event may trigger actin-independent sac-like compartment formation using unconventional mechanisms of the cellular endocytic machinery for this kind of virus internalization.

Massive endocytosis (MEND) constitutes a recently identified endocytosis mechanism that promotes the internalization of large portions of plasma membrane (25, 26). MEND is an unconventional clathrin- and dynamin-independent process whose main driving force is the coalescence of liquid-ordered membrane domains containing cholesterol and other lipids. The main characteristics of MEND are as follows: (i) it requires palmitoylation of membrane proteins (27–29); (ii) it can be regulated by Ca^2+^ activation (30); and (iii) it requires the presence of the lipid molecule phosphatidylinositol (4,5)-bisphosphate (PI(4,5)P_2_), which is phosphorylated by class I phosphoinositide 3-OH kinase (PI3K) (31–33). MEND preferentially takes place in actin-free zones and is associated with lipid-phase separation (25, 34).

Building upon previous studies that investigated viral internalization into DCs, we hypothesized that MEND could be responsible for sac-like compartment formation following viral binding to CD169. In this study, we provide evidence demonstrating that sac-like compartment formation involves extensive internalization of viral particles firmly adhered to the cell membrane that is dependent on PI3K activity and protein palmitoylation. Furthermore, we showed that cortical actin fibers transiently disappear during early stages of sac-like compartment formation. In contrast, cholesterol coalesces with the polarized virus prior to internalization and sac-like compartment formation, suggesting lipid-phase separation. In conclusion, we propose that HIV-1 exploits MEND to facilitate its internalization into DCs after binding to CD169.

## Materials and methods

### Ethics and biosafety statements

The study was approved by the institutional review board for biomedical research of Hospital Germans Trias i Pujol (HUGTiP). The participants provided written informed consent to participate in this study.

### Primary myeloid cellular model

Peripheral blood mononuclear cells (PBMCs) from HIV-seronegative donors were obtained using Ficoll-Hypaque gradient centrifugation. The monocyte population (>97% CD14^+^) was isolated using CD14-positive magnetic bead–based selection (cat. no. 130-050-201, Miltenyi Biotec). Cells were maintained at 37°C and 5% CO_2_ in RPMI medium (cat. no. 11875168, Gibco) supplemented with 10% FBS (cat. no. A5256701, Life Technologies), penicillin/streptomycin at 100 IU/ml (cat. no. 15070-022, Life Technologies), and of granulocyte-macrophage colony-stimulating factor and interleukin-4 at 1000 IU/ml (cat. no. 215-GM-500 and 204-IL-500, R&D) for seven days with replacement of media and cytokines every two days. Activated MDDCs were differentiated by culturing immature MDDCs at day five for a further two days in the presence of 100 ng/ml lipopolysaccharide (LPS, cat.no. L4391-1MG, Sigma-Aldrich) to obtain matured MDDCs.

### Generation of viral particles (VLP_HIV_)

HEK-293T/17 cells (cat. no. CRL-11268, ATCC repository) were maintained in DMEM (cat.no. 11995073, Gibco) supplemented with 10% FBS and penicillin/streptomycin at 100 IU/ml at 37°C with 8% CO_2_. VLP_HIV_ stocks were generated by transfecting 1E7 HEK-293T/17 cells with 15 µg of pHIV-Gag-eGFP plasmid (cat. no. 11468, NIH AIDS Reagent Program) using 15 µl of LipoD293 (Ver. II) reagent (cat. no. SL100668, SignaGen). After 2 days, supernatants containing VLP_HIV_ were centrifuged for 5 min at 400 xg, filtered (Millex HV, 0.45 μm; Millipore) and frozen at −80°C until use.

### Live cell imaging by confocal microscopy

MDDCs were centrifuged, washed with PBS and stained with CellTracker™ Red CMTPX Dye (cat. no. C34552, ThermoFisher). 5E5 cells diluted in 1 ml RPMI were transferred to 35-mm wells for culture imaging (cat. no. 81156, Ibidi) pretreated with poly-L-lysine 20 µg/ml (cat. no. P4832, Sigma-Aldrich). Cells were pulsed with saturating levels of VLP_HIV_ on ice for 15 min, before being washed with PBS and placed into a microscope stage incubator at 37°C and 5% CO_2_. Images were acquired using an Andor Dragonfly 505 spinning disk confocal microscope equipped with an apochromatic 100X/1.49 oil objective and a Sona 4.2 B11 sCMOS camera controlled with Fusion Software. Z-scan mode was set at a 0.2-µm XY pixel size to image the whole cell volume. Laser power was set at a minimum of 0.5% to avoid phototoxicity. Images were acquired every 2 min for 90 min. Eight fields were selected in multiposition mode to maximize the number of imaged cells.

To measure cholesterol coalescence, 5E5 MDDCs were stained for 5 min at room temperature with two lipid probes, either 75 nM of cholesterol-PEG(1k)-Abberior STAR RED dye (35, 36) or 28 nM of DPPE-Abberior STAR RED dye (cat. no. STRED-0200-1MG, Abberior GmbH). Cholesterol-PEG (1 k)-Abberior STAR RED was a gift from Prof. Christian Eggeling (Friedrich Schiller University Jena & Leibniz Institute of Photonic Technology, Jena, Germany). The probe was synthesized by fluorescent labelling of cholesterol-PEG(1000)-NH2 (PG2-AMCS, Nanocs Inc., NY, USA) with the amine reactive dye Abberior STAR RED-NHS (STRED-0002-1MG, Abberior GmbH). Cells were then washed, pulsed with VLP_HIV_, and transferred to multiwell plates (cat. no. 81156, Ibidi) for live imaging as described above.

### Inhibitory treatment of MDDCs and fixation

MDDCs stained with CellTracker™ Red CMTPX dye were treated for 2 h at 37°C with 100 nM wortmannin PI3K inhibitor (cat. no. PHZ1301, ThermoFisher), 25 µM 2-bromopalmitate palmitoyl-acyl transferase inhibitor (2-BP; cat. no. 238422, Merck), or 100 µM 5-(N-Ethyl-N-isopropyl) of the specific macropinocytosis inhibitor 5-(N-ethyl-N-isopropyl) amiloride (EIPA; cat. no. A3085, Merck). Then, MDDCs were pulsed with saturating levels of VLP_HIV_ for 15 min on ice, washed with PBS, resuspended in 1 ml RPMI, and incubated for 6 h at 37°C and 5% CO_2_. In parallel, treated cells were incubated with either labelled transferrin at 25 µg/µL (Tfn-Alexa Fluor 488; cat. no. T23366, ThermoFisher) for 30 min or with labelled recombinant cholera toxin subunit B at 1 µg/µl (Ctxβ-Alexa Fluor 488; cat. no. C34775, ThermoFisher) for 1 h. After the indicated incubation time, MDDCs were transferred to glass coverslips coated with poly-L-lysine solution (cat. no. P8920, Merck) and fixed in PFA 4% for 20 min (cat. no. P6148, Merck). PFA was then replaced with PBS-0.5% BSA w/v (cat. no. A966-506, Sigma-Aldrich) and stored at 4°C.

### Intracellular staining of fixed cells

MDDCs were treated for 1 h with permeabilization buffer (5 mg/ml BSA (cat. no. A7906 Merck) and 1 mg/ml saponin (cat. No. SAE0073, Merck). Subsequently, cells were incubated for 1 h with 20 ng/ml of anti-CD169 antibody clone #6H9 (7) and NHS conjugated with Abberior STAR RED-NHS dye (STRED-0002-1MG, Abberior GmbH). When indicated, actin fibers were stained for 1 h with 165 nM Phalloidin-Alexa Fluor 555 (cat. no. A30106, ThermoFisher) after treatment with permeabilization buffer. Nuclei were stained with 300 nM DAPI solution in PBS (cat. no. D1306, ThermoFisher) for 5 min and then washed with PBS. Coverslips were mounted onto glass slides with ProLong Glass Antifade mounting media (cat. no. P36982, ThermoFisher) and sealed with nail polish. Fixed samples were then imaged using Andor Dragonfly 505 spinning disk confocal microscope.

### Image analysis and quantification

Sac-like compartment diameter was measured by tracing a line with the line tool in Fiji/ImageJ software over the sac-like compartment in a projection of 3 XY slices comprising the sac-like compartment. To determine sac-like compartment volume, we applied the “Volume Calculator” tool in Fiji/ImageJ software using the eGFP channel signal corresponding to VLP_HIV_ in the sac-like compartment. 3D reconstruction was performed with the plugin “3D viewer” in Fiji/ImageJ software. Orthogonal views in the Z axis were obtained by tracing a line in the XY plane across the cell and applying the Reslice-Z tool in Fiji/ImageJ software.

To distinguish VLP_HIV_ distribution, we utilized a script based on the circularity of the eGFP channel. To systematize the analysis, we applied the “Subtract Background” and “Convert to Mask” commands to generate a script based on tools from Fiji/ImageJ software, which uses the cell tracker channel to determine the regions of interest (ROI) corresponding to cell boundaries. We set a minimum sac-like compartment size of 1 micron; therefore, cells with no particles were assigned to random binding. We used circularity in “Analyze Particles” to determine whether cells were polarizing VLP_HIV_ (circularity value = 0 - 0.7) or whether cells formed sac-like compartment (circularity value = 0.7 - 1). Analysis of internalization of Tfn and Ctxβ was based on the cell tracker channel. This was used to determine the ROI and analyze the mean fluorescence intensity of the green channel, which corresponds to these cargoes, inside ROIs. Image analysis scripts are provided in **Supplementary Table 1**.

We analyzed cholesterol coalescence by determining an ROI corresponding to the membrane area where VLP_HIV_ coalesces to measure the mean fluorescence intensity (MFI) of the lipid probe in the VLP_HIV_ ROI. In parallel, we determined the whole membrane ROI manually to measure the MFI of the lipid probe in the whole membrane. A ratio between the two MFI measured for each ROI was calculated as an indicator of the fold change in the cholesterol probe concentration.

We performed a pixel-by-pixel colocalization test to compare colocalization in VLP_HIV_ and CD169. This enabled us to calculate the Pearson correlation coefficient for both fluorescence signals. The Pearson correlation coefficient ranges from -1 to +1, with higher values indicating a better correlation.

### Statistical analysis

All the statistical tests were performed with GraphPad software. In the analysis of sac-like compartment size and volume, 25 cells from two different donors were measured. The results are represented using a violin plot graph, indicating median and quartiles. Average and standard deviation were calculated for volume and maximal axial size.

To analyze cholesterol coalescence, 78 cells from three different blood donors were measured. The results are represented using a violin plot graph, indicating median and quartiles. A paired t test was used to analyze the statistical significance of the increases in the cholesterol probe in the VLP_HIV_ coalescence regions of the cell membrane.

In the experiments with 2-BP, wortmannin, and EIPA inhibitory treatments, groups of at least 100 cells for each condition were counted and analyzed. MDDCs were isolated from 9, 7, and 4 blood donors, respectively. The Wilcoxon test was used to analyze the statistical significance of the changes in the phenotype distribution described for sac-like compartment formation. In control experiments to measure endocytosis of Tfn and Ctxβ upon treatment with 2-BP and wortmannin, groups of at least 100 cells for each condition were counted and analyzed. MDDCs were isolated from seven blood donors. The results are represented using a violin plot graph, indicating median and quartiles. The Wilcoxon test was performed in GraphPad to analyze the statistical significance of changes in the proportion of cells internalizing the cargoes.

## Results

### The dynamics of sac-like compartment formation do not match conventional endocytosis mechanisms

**Figure 1:**
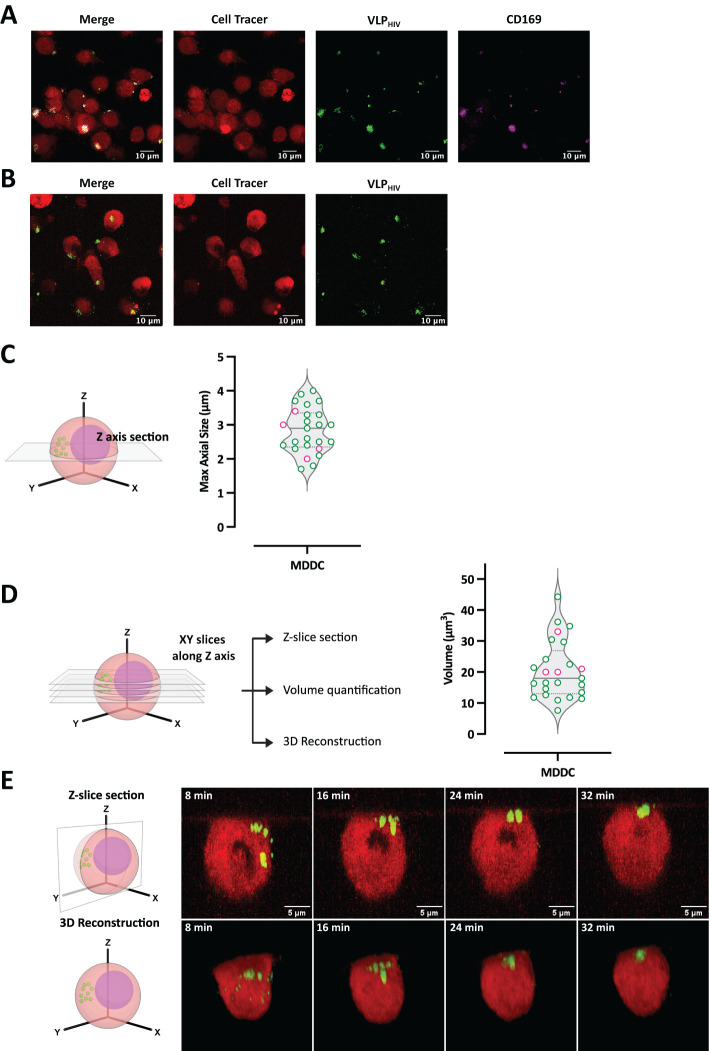
Measurement of sac-like compartment dimensions and dynamics reveals that its formation does not match conventional endocytosis pathways. MDDCs were pulsed with VLP_HIV_ (green channel) and imaged *in vivo* by live microscopy to study sac-like compartment formation and measure its size and volume. Cell volume was determined with a cell tracer fluorescent probe (red channel). **(A)** Confocal immunofluorescence of fixed MDDCs pulsed with VLP_HIV_ (green channel) and stained with anti-CD169 antibody (magenta channel) and a cell tracer (red channel). **(B)** Confocal live microscopy image of MDDCs pulsed with VLP_HIV_ (green channel) and stained with cell tracer (red channel). **(C)** Maximal axial size quantification of the sac-like compartment in 25 MDDCs from two donors. Schematic XY plane acquisition in an MDDC is presented to illustrate the maximal axial size of the sac-like compartment used. Median and quartile are shown in the graph. **(D)** Volume quantification of sac-like compartment s in 25 MDDCs from two donors (same cells as used in **Figure 1C**). Schematic view of Z-stack acquisition of multiple proximal XY planes in an MDDC is presented to illustrate the 3D images used to calculate sac-like compartment volume. Median and quartile are shown in the graph. **(E)** Sac-like compartment dynamics by live cell confocal microscopy. Images were acquired every 2 min for 70 min, and representative time points during viral polarization are shown. The top row represents projections of Z-axis slices (**Supplementary Video S1**). The bottom row represents 3D reconstruction of the same representative cell (**Supplementary Video S2**). A total of 25 MDDCs forming sac-like compartment structures were partially or totally recorded to evaluate the process. Schematic figures have been created with BioRender.

Every endocytosis mechanism produces transport intermediates with distinct sizes and morphologies. To investigate which endocytosis mechanism drives sac-like compartment formation, we measured the size, volume, and temporal dynamics of sac-like compartments in activated MDDCs. MDDCs were pulsed with HIV-1 virus-like particles expressing recombinant Gag-eGFP protein (VLP_HIV_) and imaged using confocal microscopy. MDDCs incubated in a culture chamber and imaged *in vivo* (**Figure 1A**) showed behavior similar to that of MDDCs pulsed with VLP_HIV_ and then fixed (**Figure 1B**), thus validating live cell imaging to study sac-like compartment formation. While the sac-like compartment is not perfectly spherical, we determined that the average of two donors of maximal axial size of the sac-like compartments was 2.9 ± 0.7 μm (**Figure 1C**), and the volume, measured after 3D reconstruction, was found to be 20.0 ± 9.9 μm^3^ (**Figure 1D**). These findings provide significant insight into the morphology and dimensions of sac-like compartments in MDDCs exposed to VLP_HIV_, shedding light on the intracellular dynamics of viral uptake and compartmentalization within DCs.

We conducted live-cell confocal microscopy imaging to determine whether VLP_HIV_ internalize simultaneously or accumulate after trespassing cell boundaries. Our observations from 3D reconstruction and Z-Slice projection of the corresponding microscope images revealed that bound VLP_HIV_ remained localized on the outer membrane surface until polarization in a distinct region within a timeframe of less than 5 min. Then, they internalized to form a single compartment, where all VLP_HIV_ were densely packed together (**Figure 1E**; **Supplementary Videos S1**, **S2**).

The formation of this unique compartment involves extensive internalization of VLP_HIV_ attached to the cellular membrane into a compartment in the cytoplasm. This dynamic imaging approach provides valuable insights into the temporal and spatial dynamics underlying internalization of VLP_HIV_, shedding light on the mechanisms of viral entry and compartmentalization within the cell.

### Actin cytoskeleton rearrangement participates in the biogenesis and maintenance of the sac-like compartment in MDDCs

**Figure 2:**
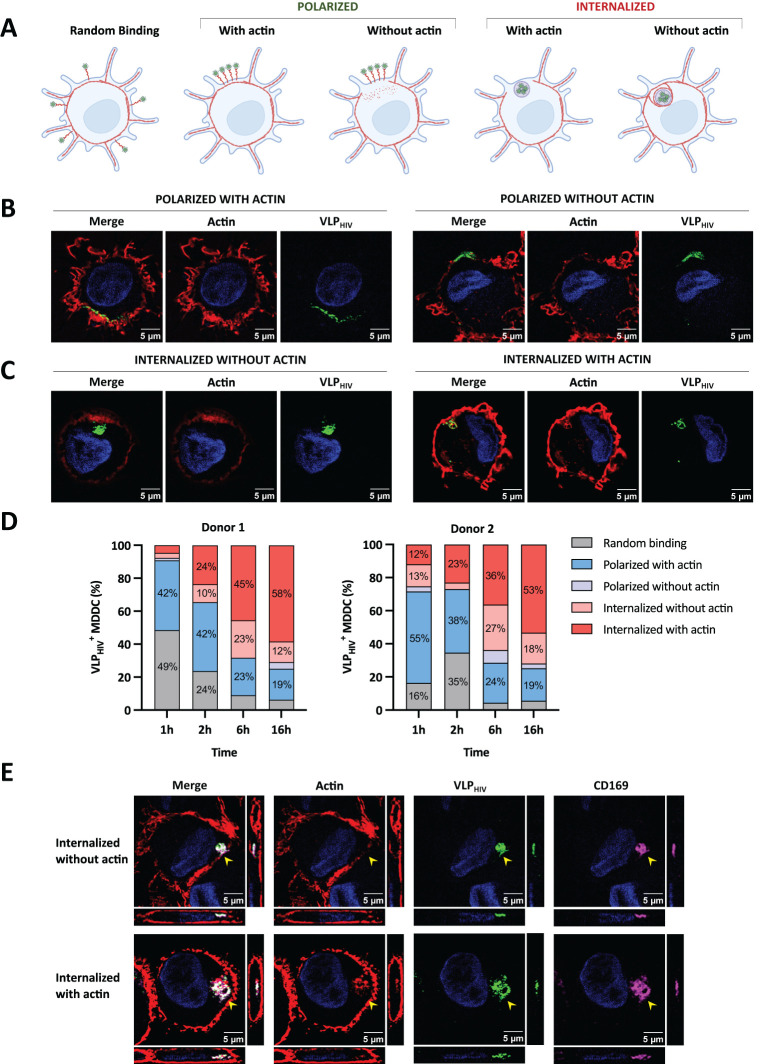
The actin cytoskeleton is essential to maintain sac-like compartment but is absent at late stages of polarization and sac-like compartment formation. MDDCs were pulsed with VLP_HIV_ (green channel) and fixed at different time points. MDDCs were stained with phalloidin-AF568 (red channel) to detect actin fibers and study their distribution during sac-like compartment formation. Cell nuclei were stained with DAPI (blue channel). **(A)** The MDDC phenotype was determined by random binding, polarization, and internalization of VLP_HIV_, as well as their potential colocalization with actin fibers. Created with BioRender.com. **(B)** Representative images of the distribution phenotype of MDDCs pulsed for 2 h with polarized VLP_HIV_. One XY slice per cell is shown. **(C)** Representative images of MDDCs pulsed for 2 h, with internalized VLP_HIV_ forming the sac-like compartment. Only one XY slice per cell is shown. **(D)** Dynamic analysis of VLP_HIV_ distribution in MDDCs from two donors classified as indicated in **Figure 2A**. Cells were pulsed with VLP_HIV_ and fixed at different time points (1 h, 2 h, 6 h, and 16 h). At least 100 cells were evaluated at each time point. The graph shows percentage values above 10% for each phenotype. **(E)** Confocal microscopy including orthogonal view of fixed MDDCs pulsed for 2 h with VLP_HIV_ and additionally stained with anti-CD169 Ab (magenta channel). The side pictures show the YZ axis slice; lower pictures show the XZ axis slice. The yellow arrow indicates the sac-like compartment.

To investigate the involvement of actin in sac-like compartment biogenesis, we pulsed MDDCs with VLP_HIV_ at different time points, fixed them, and stained actin fibers using phalloidin-AF555. This enabled us to measure changes in actin distribution and identify potential intermediate phenotypes during the sac-like compartment formation process. Based on the distribution of VLP_HIV_ and in the presence or absence of actin, we categorized five different phenotypes (**Figure 2A**). We observed that actin fibers in MDDCs were concentrated within dendrites and that the actin cortex localized beneath the plasma membrane, as compared to the rest of the cytoplasm. Polarization of VLP_HIV_ was observed in some cells, with actin fibers present in membrane-associated regions, whereas in other cells, the actin cortex was absent in regions displaying polarized VLP_HIV_ (**Figure 2B**). Some cells with internalized VLP_HIV_ did not exhibit colocalization with actin, whereas others displayed fully formed sac-like compartment that colocalized with actin fibers (**Figure 2C**).

When we quantified the dynamic incorporation of VLP_HIV_ and its colocalization with actin fibers in MDDCs from different donors, we observed that VLP_HIV_ polarized in a single pole phenotype is more abundant than a sac-like compartment fully formed by internalized VLP_HIV_ at early time points (**Figure 2D**).

The proportion of cells with VLP_HIV_ internalized in sac-like compartments colocalizing with actin increased from 10% at earlier time points to over 50% of total evaluated cells after 16 h of incubation. This suggests that actin colocalization represents the final stage in the sac-like compartment formation process. Concurrently, the polarized phenotype colocalizing with actin decreased from approximately 50% to less than 20% of total evaluated cells. Interestingly, phenotypes where actin does not colocalize with VLP_HIV_ were more frequent at intermediate time points, suggesting they are transient states during sac-like compartment formation. Moreover, the orthogonal view of sac-like compartments lacking actin colocalization revealed discontinuities in the actin cortex, forming channel-like structures (**Figure 2E**). Colocalization analysis of CD169 and VLP_HIV_ in these structures showed a Pearson correlation index of 0.85, indicating a high degree of colocalization degree in both (**Figure 2E**). All sac-like compartments phenotypes exhibited colocalization with CD169 expression (**Figure 2E**), thus reinforcing the role of CD169 in mediating viral uptake. To further validate the abundance of these phenotypes, MDDCs from four additional blood donors were incubated with VLP_HIV_ for 2h to maximize the visualization of intermediate polarized and internalized phenotypes (**Supplementary Figure S1**).

In conclusion, these data suggest that the actin cytoskeleton plays a role in the early stages of polarization of VLP_HIV_, making it crucial to preserve the structural integrity of sac-like compartments in the final phase. The data also indicates that rearrangement of cortical actin accompanies internalization of HIV at early stages of sac-like compartment formation, suggesting the involvement of a MEND-like mechanism.

### Plasma membrane cholesterol cotraffics with viral particles and accumulates within the sac-like compartment

**Figure 3:**
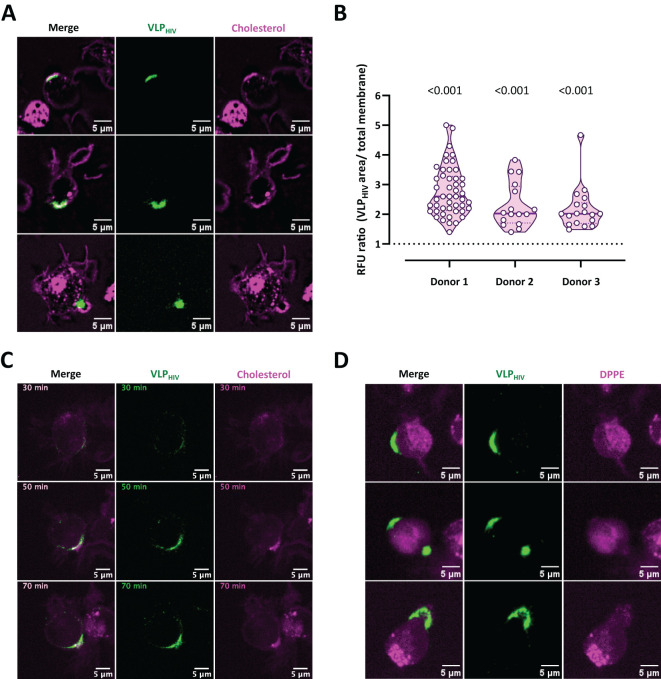
Cholesterol cotraffics during VLP_HIV_ polarization and accumulates within sac-like compartment. MDDCs from three donors were incubated with fluorescently labelled cholesterol or phospholipid DPPE probes (magenta channel), pulsed for 1 h with VLP_HIV_ (green channel), and analyzed using live cell confocal microscopy. **(A)** Representative images of three different fields showing MDDCs incubated with the cholesterol probe during sac-like compartment formation. **(B)** Quantification of cholesterol fluorescence intensity in membrane areas in 78 MDDCs from three different donors. The relative fluorescence units (RFU) ratio was calculated by dividing fluorescence intensity at the VLP_HIV_ polarization region by the fluorescence of the whole membrane. The horizontal bar indicates the average ratio for each donor. The t test was applied to evaluate statistical significance. The P-value for each donor is shown on the graph. Only significant P-values are shown. **(C)** Dynamics of cholesterol accumulation during polarization of VLP_HIV_, as shown in **Figure 3A**. Images were acquired every 5 min for 95 min. Representative time points during viral polarization are shown. (**Supplementary Video S3**). **(D)** Representative images of three MDDCs incubated with the phospholipid DPPE probe during sac-like compartment formation.

We subsequently used live confocal microscopy with a fluorescent cholesterol analogue probe to investigate whether the MEND pathway accompanied by cholesterol coalescence could contribute to sac-like compartment formation (**Figure 3**). Remarkably, the fluorescent signal of the cholesterol analogue increased notably in cell membrane regions containing VLP_HIV_, exhibiting both polarized and internalized distribution phenotypes (**Figure 3A**). We quantified the fluorescence intensity of this cholesterol probe in membrane regions where viral particles accumulated before sac-like compartment formation and compared it to the average fluorescence intensity of the cellular membrane. Our analysis focused solely on cells displaying the polarization phenotype, as sac-like compartment formation involves membrane folding that enhances the cholesterol fluorescence signal (**Figure 3B**). We observed that the cholesterol fluorescence intensity at regions where sac-like compartments were forming was twice as high as in the rest of the membrane. This trend persisted over time, confirming the redistribution and coalescence of cholesterol, with accumulation of VLP_HIV_ in the cellular membrane (**Figure 3C**; **Supplementary Video S3**).

As a control, we simultaneously used a fluorescent dipalmitoyl phosphatidylethanolamine analogue probe (DPPE) to assess the lipid bilayer independently of cholesterol lipid rafts (**Figure 3D**). In contrast to the cholesterol analogue, this lipid molecule exhibited a uniform distribution in the cell membrane, with no localized increase in membrane regions where VLP_HIV_ accumulated.

These findings demonstrate that cholesterol coalescence in the cell membrane is associated with the accumulation of VLP_HIV_ prior to sac-like compartment formation, supporting the notion that MEND mechanisms play a crucial role in sac-like compartment formation in myeloid cells.

### Inhibition of palmitoylation arrests sac-like compartment formation without affecting CD169 expression

**Figure 4:**
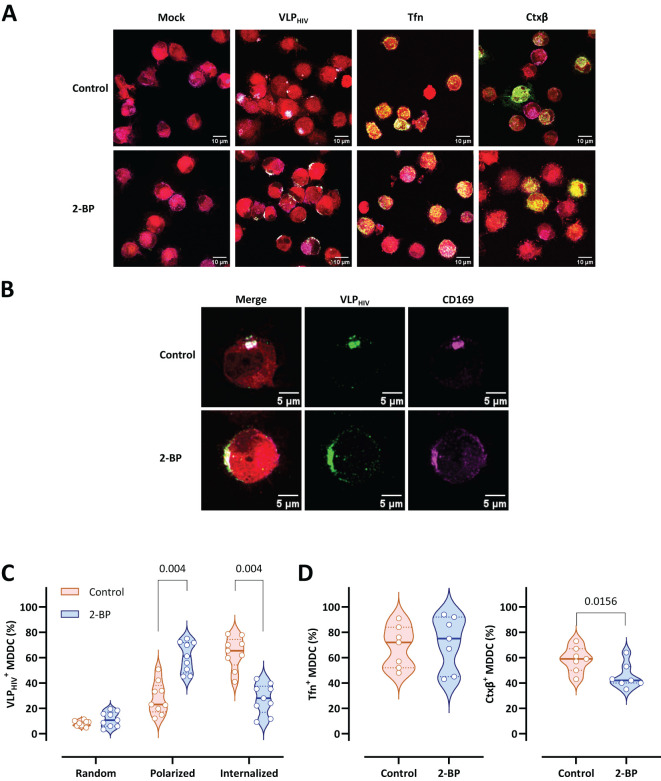
Palmitoylation inhibition arrests VLP_HIV_ polarization and sac-like compartment formation. MDDCs from nine donors were treated for 2 h with 2-bromopalmitate (2-BP) and pulsed for 4 h with VLP_HIV_ (green channel). In control assays, treated MDDCs were pulsed with transferrin (Tfn) for 30 min or with cholera toxin subunit β (Ctxβ) for 1 h (green channel). The cell cytoplasm was stained with a fluorescent cell tracer probe (red channel). Fixed cells were stained with anti-CD169 Ab (magenta channel). More than 100 cells from each donor were evaluated for each condition. **(A)** Comparative confocal microscopy images of MDDCs pulsed with VLP_HIV_, Tfn, or Ctxβ upon treatment with 2-BP. Images show a projection of five to ten Z-slices corresponding to the equatorial area of cargo internalization. **(B)** Representative cell from **Figure 4A**. **(C)** Quantitative analysis of VLP_HIV_ distribution upon treatment with 2-BP according to three different phenotypes: random binding, polarized, and internalized. 4 different experiments were performed using cells from 9 different blood donors. Each dot indicates the percentage of cells for each phenotype for single donors. Violin plots indicate the average percentage of each phenotype for all donors. Median and quartiles are shown in the violin plot. The Wilcoxon test was applied to evaluate statistical significance. The P-value for each phenotype is shown in the graph. Only significant P-values are shown. **(D)** Quantitative analysis of internalization of Tfn and Ctxβ in MDDCs upon treatment with 2-BP. Each dot indicates the percentage of cells that have internalized Tfn or Ctxβ for single donors. Violin plots indicate the average percentage abundance of each phenotype for all donors. Median and quartiles are represented in the violin plot. The Wilcoxon test was applied to evaluate statistical significance. The P-value is shown in the graph. Only significant P-values are shown.

In accordance with the current definition of MEND, protein palmitoylation initiates the mechanism that facilitates protein coalescence and activates MEND. To assess the significance of protein palmitoylation in sac-like compartment formation in MDDCs, we used the palmitoylation inhibitor 2-bromopalmitate (2-BP). 2-BP abrogates the internalization of VLP_HIV_ into the sac-like compartment, halting them at the polarization stage on the cell membrane (**Figure 4A**). The potential impact of 2-BP on conventional endocytosis pathways was also investigated by separately pulsing MDDCs with transferrin (Tfn) and cholera toxin subunit β (Ctxβ)–labelled reporters, which are commonly used cargoes for endocytosis assays. No obvious differences were observed in the cell uptake of Tfn or Ctxβ after 2-BP treatment, thus ruling the role of palmitoylation in this endocytosis process (**Figure 4A**). Interestingly, zoomed-in representative cells revealed that VLP_HIV_ failed to complete internalization upon treatment with 2-BP. Additionally, CD169 expression remained unaltered following treatment with 2-BP, indicating that arrest of uptake cannot be attributed to a lack of CD169 (**Figure 4B**).

Quantitative data demonstrated that the proportion of MDDCs arrested at the polarization stage increased from 25% to 60% following treatment with 2-BP compared to untreated MDDCs (**Figure 4C**). Inversely, treatment with 2-BP drastically reduced sac-like compartment formation (25%) compared to controls (60%), arresting the VLP_HIV_ uptake process at the polarization stage (**Figure 4C**). MDDCs treated with 2-BP were concurrently pulsed with Tfn- and Ctxβ-labelled reporters to evaluate any potential side effects on classical endocytosis pathways (**Figure 4D**). We observed that the internalization patterns of transferrin remained unaltered upon treatment and that internalization of Ctxβ is minimally affected compared with sac-like compartment formation.

In conclusion, these data suggest that sac-like compartment formation is dependent on protein palmitoylation, which is another essential regulatory mechanism for MEND.

### Inhibition of the PI3K metabolic pathway halts sac-like compartment formation without affecting CD169 expression

**Figure 5:**
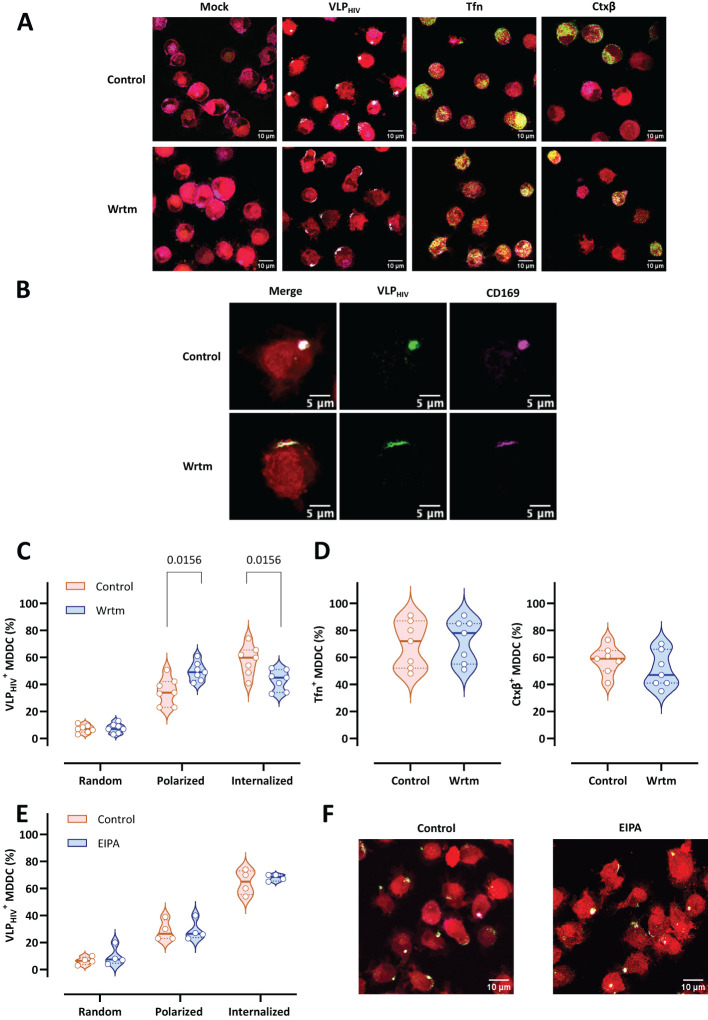
Inhibition of the PI3K metabolic pathway arrests VLP_HIV_ polarization and sac-like compartment formation. MDDCs from seven donors were treated with wortmannin (PI3K inhibitor named Wrtm) for 2 h and pulsed for 4 h with VLP_HIV_ (green channel). In control assays, treated MDDCs were pulsed with Tfn for 30 min or with Ctxβ for 1 h (green channel). The cell cytoplasm was stained with a fluorescent cell tracer probe (red channel). Fixed cells were stained with anti-CD169 Ab (magenta channel). More than 100 cells from each donor were counted for each condition. **(A)** Comparative confocal microscopy images of MDDCs pulsed with VLP_HIV_, Tfn, or Ctxβ upon treatment with wortmannin. Images show a projection of five to ten Z-slices corresponding to the equatorial area of cargo internalization. **(B)** Representative cell from **Figure 5A**. **(C)** Quantitative analysis of VLP_HIV_ distribution upon treatment with wortmannin according to 3 different phenotypes: random binding, polarized, and internalized. Each dot indicates the percentage of cells for each phenotype for single donors. Violin plots indicate the average percentage of each phenotype for all donors. Median and quartiles are represented in the violin plot. The Wilcoxon test was applied to evaluate statistical significance. Only significant P-values are shown. **(D)** Quantitative analysis of internalization of Tfn and Ctxβ in MDDCs from five donors upon treatment with wortmannin. The Wilcoxon test was applied to evaluate statistical significance. Each dot indicates the percentage of cells that have internalized Tfn or Ctxβ for single donors. Violin plots indicate the average percentage abundance of each phenotype for all donors. Median and quartiles are shown in the violin plot. Only significant p-values are shown in the graph. Only significant P-values are shown. **(E)** Quantitative analysis of VLP_HIV_ distribution in MDDCs from four donors upon treatment with the macropinocytosis inhibitor EIPA, as evaluated in **Figure 5B**. Each dot indicates the percentage of cells for each phenotype for single donors. Violin plots indicate the average percentage of each phenotype for all donors. Median and quartiles are shown in the violin plot. Only significant P-values are shown. **(F)** Comparative confocal microscopy images of MDDCs pulsed with VLP_HIV_ upon treatment with EIPA. Images show a projection of five to ten Z-slices corresponding to the equatorial area of cargo internalization.

To assess the reliance of sac-like compartment formation on (PI(4,5)P_2_) phosphorylation by PI3K, MDDCs from various donors were treated with the PI3K paninhibitor wortmannin prior to the VLP_HIV_ pulse. Wortmannin halts sac-like compartment formation at the VLP_HIV_ polarization stage in the cell membrane (**Figure 5A**). To exclude side effects of wortmannin on conventional endocytosis mechanisms, cell uptake of Tfn or Ctxβ was assessed as described above, with no evident differences observed for either after treatment with wortmannin (**Figure 5A**). This incomplete VLP_HIV_ polarization phenotype is characterized by a semicircular distribution of VLP_HIV_, which do not converge at a single membrane region for internalization. Importantly, treatment with wortmannin does not interfere with either CD169 expression or the attachment of VLP_HIV_ (**Figure 5B**).

Quantitatively, the proportion of cells arrested at the polarization stage after treatment with wortmannin increased from 30% to 50% (**Figure 5C**). Moreover, the percentage of MDDCs with internalized VLP_HIV_ forming sac-like compartments decreased from over 60% to 40%, halting the VLP_HIV_ uptake process at the polarization stage. As a control, we evaluated internalization of Tfn and Ctxβ, which was comparable between wortmannin-treated and untreated MDCCs, indicating that wortmannin does not impair conventional endocytosis pathways in this cellular model (**Figure 5D**).

As PI3K metabolism also plays an important role in macropinocytosis, we used the specific macropinocytosis inhibitor 5-(N-ethyl-N-isopropyl) amiloride (EIPA) to evaluate whether the effect observed by wortmannin could be explained by inhibition of macropinocytosis (**Figures 5E, F**). EIPA functions by inhibiting Na^+^/H^+^ exchangers, thereby preventing the activation of intracellular macropinocytosis regulators. Pretreatment with EIPA did not show any significant effect on sac-like compartment formation, confirming that macropinocytosis is not involved in sac-like compartment formation.

Collectively, these findings suggest that when PI3K metabolism is inhibited, internalization of virus in MDDCs is diminished at the polarization stage. This inhibition of PI3K has been reported to be an additional regulatory mechanism of MEND.

## Discussion

Understanding the mechanism underlying internalization of the enveloped virus into myeloid cells plays a critical role in the development of new therapeutical strategies to halt viral dissemination throughout the body. Previous studies employing endocytosis inhibitor treatments have concluded that this route of entry for HIV-1 is clathrin-independent and cholesterol-dependent (1, 24), while the involvement of macropinocytosis required further investigation (14). Since the actin cytoskeleton is dispensable for migration and internalization of viral particles into the sac-like compartment in myeloid cells (25, 34) and macropinocytosis relies on actin fibers to mediate membrane folding (21, 25, 26), we propose that macropinocytosis is not the primary driver mechanism during sac-like compartment formation.

CD169 plays a significant role as an attachment receptor during entry of HIV-1 into activated myeloid cells, because its interaction prompts internalization of viral particles into the sac-like compartment, enhancing subsequent viral trans-infection to target cells (6). This phenomenon has been further demonstrated using blocking antibodies targeting CD169, which prevent viral uptake, and by the use of point mutants in the ganglioside-binding domain of this cellular receptor (5–7, 9, 10, 14, 15, 37–39). Although there is limited knowledge about which interactors of CD169 might participate in a specific endocytosis mechanism, this study provides novel insights into the CD169-mediated uptake of HIV-1 by myeloid cells and, potentially, of other highly pathogenic enveloped viruses, providing us with new therapeutic targets to halt their dissemination throughout the body.

To facilitate the characterization of this process *in vitro*, we used an MDDC system derived from CD14^+^ monocytes isolated from peripheral blood. This system has been validated as an appropriated DC model for studying the role of myeloid cells in HIV-1 *trans*-infection from sac-like compartments upon binding to CD169 (1, 3, 13). *Ex vivo* studies have demonstrated that cervical HLA-DR^+^ CD14^+^ CD11c^+^ DCs and tonsillar CD11c^+^ BDCA1^+^ DCs can capture and *trans*-infect HIV-1 via CD169. Moreover, sac-like compartments have been observed *in vivo* in cervical tissue from infected patients (2, 15). Recent studies have shown that MDMs and MDDCs from dermal tissue cultured *ex vivo* express CD169, which partially mediates HIV-1 capture (16). A recent study evaluating primary human DC subpopulations showed that only a subset constitutively expressing CD169 is susceptible to HIV-1 infection in a CD169–dependent manner, thus highlighting the role of this attachment receptor in viral uptake (38). Other studies have assessed HIV-1 *trans*-infection into CD4^+^ T cells in alternative cellular models with limited CD169 expression, indicating that other host factors may be relevant during the process (16, 17, 40). Thus, despite their limitations, *in vitro* differentiated MDDCs can serve as a robust model for studying sac-like compartment biogenesis, with the potential to transfer this knowledge to other experimental models of dendritic cells that more closely resemble bona fide DCs (41).

Endocytosis pathways, which typically generate transport intermediates with varying sizes and morphologies, include membrane protrusions that elongate to envelop cargo during phagocytosis (21, 42). In our study, we investigated VLP_HIV_ uptake in MDDCs using live-cell confocal microscopy to quantitatively describe sac-like compartment dimensions. Our 3D reconstruction data clearly demonstrate that VLP_HIV_ migrate to a single pole prior to sac-like compartment formation. Following polarization, VLP_HIV_ internalized into a single compartment, with no evidence of smaller endosomal structures coalescing after internalization, as typically observed in the endosome recycling pathway, suggesting that a substantial amount of cell membrane rapidly folds back into the cell. Massive membrane folding is a characteristic compatible with MEND, as more than 50% of the plasma membrane can be internalized in experimental models^25.^ In this regard, previous studies have reported that the sac-like compartment maintains continuity with the cell membrane (11, 14), implying substantial folding of the cell membrane into cell cytoplasm. We also characterized the morphology and dimensions of the unified sac-like compartment and found that, interestingly, their average dimensions exceeded those typically observed in endosomes generated through conventional endocytosis pathways (43). Although MEND has been reported to produce vesicles smaller than 250 nm, in contrast to our results, other cell models have shown palmitoylation-dependent endocytosis processes that generate vesicles ranging from 2 μm to 5 μm, suggesting that MEND could produce vesicles of greater sizes (25, 43). In our case, we propose that VLP_HIV_ utilize MEND mechanisms to form the sac-like compartment instead of triggering a canonical endocytosis process, as sac-like compartment do not incorporate into the endosome pathway and maintain viral particles inside for several hours (1). The fact that the sac-like compartment is surrounded by an actin cytoskeleton may impede the transport of the VCC to the endolysosomal pathway, thus preserving the infectivity of trapped viruses.

While the actin cytoskeleton is known to participate in various endocytosis mechanisms (43), its specific role during sac-like compartment formation remains controversial (14, 23). The role of actin in sac-like compartment formation was previously reported to be essential, based on the inhibitory effect of latrunculin-A, a compound that disrupts actin cytoskeleton organization (14). However, this effect could be explained by the dependency of nanoscale CD169 organization on actin fibers, which increases its avidity for ligands (23). Therefore, actin deregulation may disrupt CD169 nanoscale clusters, hindering VLP_HIV_ binding to CD169 and sac-like compartment formation. Thus, actin appears to be essential at the initial stages of sac-like compartment formation. However, after viral binding, the activity of the actin regulators pERM and formin must be abrogated to enable free diffusion of the CD169-virion complex for polarization and internalization (23). We propose a dual role for actin during sac-like compartment formation, where it is essential for viral binding but dispensable during polarization and sac-like compartment formation. Our findings corroborate this hypothesis, as actin fibers went undetected in some MDDCs with polarized VLP_HIV_ and internalized into the sac-like compartment. However, this phenotype did not increase its frequency over time, suggesting that such phenotypes are transient states during the sac-like compartment formation process. Disappearance of actin fibers during sac-like compartment formation correlates with a decrease in the activity of pERM and formin during VLP_HIV_ polarization (23). In contrast, the sac-like compartment increases colocalization with actin over time, indicating that colocalization is the final stage of the sac-like compartment formation process to maintain its structure, in the same way as actin fibers maintain cell shape and organelle structure. Although CD169 is present at every step of the sac-like compartment formation process regardless of the presence of actin, it is still unknown how the binding of viral particles to this receptor triggers actin remodeling and what other potential host factors could bind to actin molecules.

Previous studies utilizing PI3K inhibitors such as LY294002 in MDDCs have shown an arrest in sac-like compartment formation under confocal microscopy, resulting in the random distribution of VLP_HIV_ across the membrane (44). This finding is consistent with our results, which demonstrated the dependency of sac-like compartment formation on PI3K activity. As PI3K also regulates macropinocytosis, we used the specific macropinocytosis inhibitor EIPA before pulsing cells with VLP_HIV_ and showed that this endocytosis mechanism is not involved in sac-like compartment formation, in line with previous observations (14). Anyway, PI3K inhibitors target a central component in cell signaling pathways, making it challenging to delineate the downstream pathway responsible for sac-like compartment internalization and MEND signaling. Nonetheless, this approach provides a novel experimental model for investigating MEND activation under physiological stimuli, as opposed to restrictive experimental conditions implying high concentrations of Ca^2+^, CoA, albumin, or palmitate (25). Consequently, MDDCs constitute a promising experimental model for advancing comprehension of MEND regulation and triggering mechanisms.

Cholesterol coalescence plays a pivotal role in membrane phase separation processes, serving as a primary driving force of the MEND mechanism. In our findings we present the initial evidence of cholesterol coalescence in MDDCs associated with MEND, as observed in an experimental model using a fluorescent cholesterol probe. While the presence of cholesterol in the cell membrane is crucial for viral *trans*-infection, previous studies assessed its impact on compromised cell viability without evaluating its distribution across the membrane during the sac-like compartment formation process (1). Our study demonstrated that a cholesterol probe effectively labels the cell membrane in MDDCs without affecting cell viability, enabling real-time visualization of cholesterol coalescence alongside VLP_HIV_ within MDDCs.

An association has been observed between palmitoylated proteins and cholesterol raft domains in the cell membrane, leading to their coalescence and initiating membrane phase separation. Thus, protein palmitoylation is a key regulatory mechanism in MEND. Inhibition of palmitoyl-acyl transferase proteins such as DHHC2 and DHHC5 has been shown to decrease MEND activity in various studies (45–49). In our research, chemical inhibition of these proteins with the inhibitor 2-BP significantly reduces sac-like compartment formation, providing further evidence of the connection between MEND and sac-like compartment formation. Given that palmitic acid residues are saturated fatty acids, they may contribute to the change in lipid density when palmitoylated proteins accumulate at specific sites (50–53). While CD169 is predicted to have a palmitoylated cysteine position in its cytoplasmatic tail, further investigation is needed to determine whether CD169 is palmitoylated following VLP_HIV_ binding, or if alternative host factors interacting with CD169 could undergo palmitoylation.

The sac-like compartment shares characteristics with VCCs in macrophages, which contain *de novo* synthesized virions (18, 19). Recent investigations into VCC formation in macrophages suggest the involvement of an alternative endocytosis pathway known as CLIC/GEEC, in HIV-1 uptake (20). Interestingly, MEND and CLIC/GEEC pathways are similar, with variable dependence on actin remodeling and lipid ordering, suggesting that they may form a continuum of related mechanisms (25). Therefore, the formation of VCC in macrophages and sac-like compartments in dendritic cells likely involves highly related endocytic mechanisms.

In conclusion, our findings demonstrate extensive plasma membrane invagination and cholesterol coalescence, suggesting lipid-phase separation during internalization of HIV-1 into myeloid cells, which is potentially triggered upon viral binding to CD169. Additionally, the study revealed transient depolarization of cortical actin during sac-like compartment formation, as well as the involvement of regulatory mechanisms such as PIP metabolism and protein palmitoylation. These key processes align with the definition of MEND. The new knowledge we report sheds light on the mechanisms underlying viral uptake into myeloid cells, which is crucial for understanding viral spread throughout the body. These insights open new possibilities for developing novel therapeutic strategies to combat enveloped viruses that use CD169 as an attachment receptor.

## Data Availability

The raw data supporting the conclusions of this article will be made available by the authors, without undue reservation.

## Supplementary material

Supplementary Video 1 Z-slice confocal live microscopy video to illustrate VLP_HIV_ uptake by MDDCs and sac-like compartment formation. Cells were plated onto poly-L-lysine plastic culture dishes, mounted in a microscope chamber at 37°C, 5% CO_2_, and imaged at 2-min intervals using confocal fluorescence microscopy immediately after the VLP_HIV_ pulse (green channel). Cell volume was determined with a cell tracer fluorescent probe (red channel). The video was rendered using the reslice tool in FIJI.

Supplementary Video 2 3D reconstruction confocal live microscopy video to illustrate VLP_HIV_ uptake by MDDCs and sac-like compartment formation. Cells were plated onto poly-L-lysine plastic culture dishes, mounted in a microscope chamber at 37°C, 5% CO_2_, and imaged at 2-min intervals using confocal fluorescence microscopy immediately after the VLP_HIV_ pulse (green channel). The cell volume was determined with a cell tracer fluorescent probe (red channel). The video was rendered using the 3D reconstruction in FIJI.

Supplementary Video 3 Confocal live microscopy video to illustrate cholesterol coalescence during VLP_HIV_ uptake and polarization by MDDCs. Cells incubated with a fluorescent cholesterol probe (purple channel) were plated onto poly-L-lysine plastic culture dishes, mounted in a microscope chamber at 37°C, 5% CO_2_, and imaged at 5-min intervals using confocal fluorescence microscopy immediately after the VLP_HIV_ pulse (green channel).

Supplementary Figure 1 VLP_HIV_ distribution depending on actin colocalization in MDDCs from six donors. Analysis of VLP_HIV_ distribution in MDDCs from six donors classified as indicated in **Figure 2A**. Cells were pulsed with VLP_HIV_ and fixed at 2h. At least 20 cells were evaluated at each time point. The graph shows percentage values above 10% for each phenotype.
