# Supplementary figures and images for: Massive endocytosis mechanisms are involved in uptake of HIV-1 particles by monocyte-derived dendritic cells
Source: Front Immunol. 2025 Jan 10;15:1505840. doi: 10.3389/fimmu.2024.1505840 (PMC11757119; doi:10.3389/fimmu.2024.1505840)

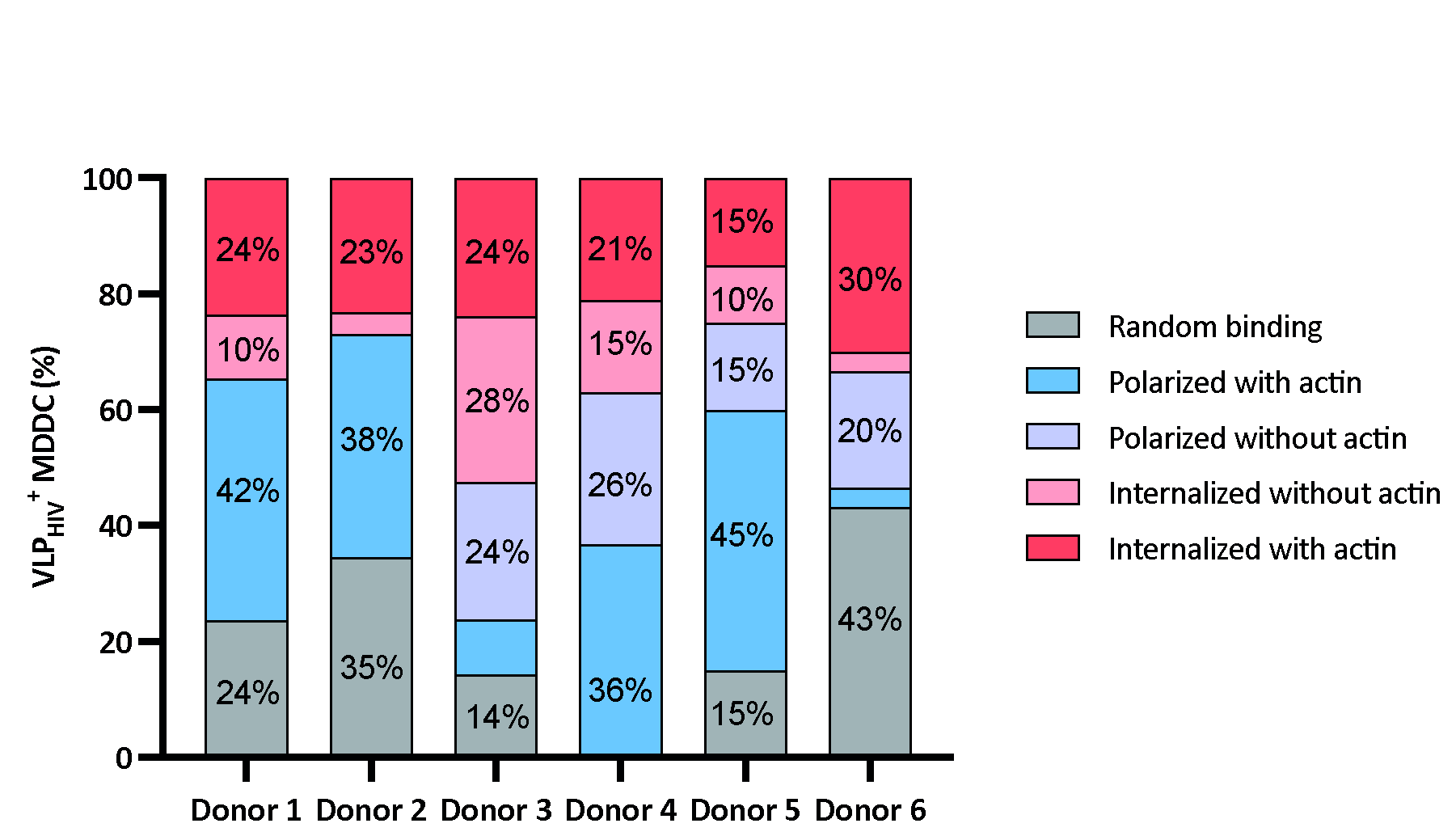

Supplement: Supplementary Figure 1 — VLPHIV distribution depending on actin colocalization in MDDCs from six donors. Analysis of VLPHIV distribution in MDDCs from six donors classified as indicated in Figure 2A . Cells were pulsed with VLPHIV and fixed at 2h. At least 20 cells were evaluated at each time point. The graph shows percentage values above 10% for each phenotype. [file Image1.tif]
