# Supplementary material for: Massive endocytosis mechanisms are involved in uptake of HIV-1 particles by monocyte-derived dendritic cells
Source: Front Immunol. 2025 Jan 10;15:1505840. doi: 10.3389/fimmu.2024.1505840 (PMC11757119; doi:10.3389/fimmu.2024.1505840)
Supplement: Supplementary file 5 [file DataSheet1.pdf]

Supplemental Table 1. Automated analysis procedure for cell counting, VLP_{HIV} distribution and quantification of endocytosis of control cargoes Tfn and Ctxβ. Threshold min and max values should be set for each analysis taking the corresponding mock condition as a reference.

Cell count	
STEP 1	run("Duplicate...", "duplicate channels=1");
STEP 2	setThreshold(500, 65535, "raw");
STEP 3	setOption("BlackBackground", true);
STEP 4	run("Convert to Mask");
STEP 5	run("Gaussian Blur...", "sigma=2");
STEP 6	run("Convert to Mask");
STEP 7	run("Watershed");
STEP 8	run("Create Selection");
STEP 9	roiManager("Add");
STEP 10	roiManager("Select", 0);
STEP 11	roiManager("Rename", "Cells");
STEP 12	roiManager("Select", 0);
STEP 13	run("Analyze Particles...", "size=50-Infinity circularity=0-1 display clear include overlay composite");
**Sac-containing compartment analysis**	
STEP 1	run("Enhance Contrast", "saturated=0.35");
STEP 2	Stack.setChannel(1);
STEP 3	run("Magenta");
STEP 4	run("Enhance Contrast", "saturated=0.35");
STEP 5	Stack.setChannel(2);
STEP 6	run("Red");
STEP 7	run("Enhance Contrast", "saturated=0.35");
STEP 8	Stack.setChannel(3);
STEP 9	run("Green");
STEP 10	run("Enhance Contrast", "saturated=0.35");
STEP 11	Stack.setChannel(4);
STEP 12	run("Blue");
STEP 13	Stack.setDisplayMode("composite");
STEP 14	run("Z Project...", "start=X stop=Y projection=[Max Intensity]");
STEP 15	run("Duplicate...", "duplicate channels=2");
STEP 16	setThreshold(800, 65535, "raw");
STEP 17	setOption("BlackBackground", true);
STEP 18	run("Convert to Mask");
STEP 19	run("Gaussian Blur...", "sigma=2");
STEP 20	run("Convert to Mask");
STEP 21	run("Watershed");
STEP 22	run("Create Selection");
STEP 23	roiManager("Add");
STEP 24	roiManager("Select", 0);
STEP 25	roiManager("Rename", "Cells");

STEP 26	close()
STEP 27	run("Duplicate...", "duplicate channels=3");
STEP 31	setThreshold(3073, 65535, "raw");
STEP 32	run("Convert to Mask");
STEP 33	run("Gaussian Blur...", "sigma=2");
STEP 34	run("Convert to Mask");
STEP 35	run("Create Selection");
STEP 36	roiManager("Add");
STEP 37	roiManager("Select", 1);
STEP 38	roiManager("Rename", "HIV");
STEP 39	roiManager("Select", 1);
STEP 40	roiManager("Select", 0);
STEP 41	roiManager("Select", newArray(0,1));
STEP 42	roiManager("AND");
STEP 43	roiManager("Add");
STEP 44	roiManager("Select", 2);
STEP 45	roiManager("Rename", "SCC");
STEP 46	//Circularity: 0-0.7 for polarized phenotype; 0.7-1 for sac-containing compartment
STEP 47	//Size: 2-Infinity for polarized phenotype; 4-Infinity for sac-contaning compartment
STEP 48	run("Analyze Particles...", "size=2-Infinity circularity=0-0.7 display clear include overlay composite");
**Analysis of transferrin or Ctxβ**	
Step 1	run("Duplicate...", "duplicate channels=1");
Step 2	run("Auto Threshold", "method=Mean white");
Step 3	setOption("BlackBackground", true);
Step 4	run("Convert to Mask");
Step 5	run("Watershed");
Step 6	close();
Step 7	close("Results")
Step 8	run("Duplicate...", "duplicate channels=2");
Step 9	//TFN 200-800 //CTXB 120-500
Step 10	setMinAndMax(50,550);
Step 11	setAutoThreshold("Default dark no-reset");
Step 12	setOption("BlackBackground", true);
Step 13	run("Convert to Mask");
Step 14	run("Select All");
Step 15	roiManager("Add");
Step 16	roiManager("Measure");
Step 17	run("Analyze Particles...", "size=20-5000 show=Overlay display clear include add");
